# Effects of Dating Matters® on Sexual Violence and Sexual Harassment Outcomes among Middle School Youth: a Cluster-Randomized Controlled Trial

**DOI:** 10.1007/s11121-020-01152-0

**Published:** 2020-08-26

**Authors:** Sarah DeGue, Phyllis Holditch Niolon, Lianne Fuino Estefan, Allison J. Tracy, Vi D. Le, Alana M. Vivolo-Kantor, Todd D. Little, Natasha E. Latzman, Andra Tharp, Kyle M. Lang, Bruce Taylor

**Affiliations:** 1 Division of Violence Prevention, National Center for Injury Prevention and Control, Centers for Disease Control and Prevention, 4770 Buford Highway NE, MS-F63, Atlanta, GA 30341 USA; 2 2M Research, LLC, Arlington, TX USA; 3 Institute for Measurement, Methodology, Analysis and Policy, Texas Tech University, Lubbock, TX USA; 4 NORC at the University of Chicago, Chicago, IL USA

**Keywords:** Dating Matters, Sexual violence, Sexual harassment, Teen dating violence, Prevention

## Abstract

Sexual violence (SV), including sexual harassment (SH), is a significant public health problem affecting adolescent health and well-being. This study extends prior research by evaluating the effectiveness of a comprehensive teen dating violence prevention model, Dating Matters, on SV and SH perpetration and victimization, inclusive of any victim-perpetrator relationship, among middle school students. Dating Matters includes classroom-delivered programs for youth in 6th, 7th, and 8th grades; community-based programs for parents; a youth communications program; training for educators; and community-level activities. Middle schools in four urban areas in the USA were randomly assigned to receive Dating Matters (DM, *N* = 22) or a standard-of-care intervention (SC, *N* = 24) over four consecutive school years (2012–2016). The analytic sample included two cohorts who entered the study in 6th grade and completed 8th grade by the end of the study allowing for full exposure to Dating Matters (DM: *N* = 1662; SC: *N* = 1639; 53% female; 50% black, non-Hispanic; 6 waves of data collection for each cohort). Structural equation modeling was employed with multiple imputation to account for missing data. Dating Matters was associated with significant reductions in SV and SH perpetration and victimization scores in most—but not all—sex/cohort groups by the end of 8th grade relative to an evidence-based TDV prevention program. On average, students receiving Dating Matters scored 6% lower on SV perpetration, 3% lower on SV victimization, 4% lower on SH perpetration, and 8% lower on SH victimization by the end of middle school than students receiving an evidence-based violence prevention program. Overall, Dating Matters shows promise for reducing SV and SH, occurring both within and outside dating relationships, through middle school. Clinicaltrials.gov Identifier: NCT01672541

**Electronic supplementary material:**

The online version of this article (10.1007/s11121-020-01152-0) contains supplementary material, which is available to authorized users.

Sexual violence^1^—including sexual dating violence and sexual harassment—affects millions of US teens each year with harmful effects on their short- and long-term health, safety, and well-being (Ackard et al. 2007; Chiodo et al. 2009; Exner-Cortens et al. 2013). About 1 in 14 high school students (and 1 in 9 high school girls) have experienced rape at some point in their life, and 1 in 10 report some form of sexual violence (SV) victimization—defined as being forced to do “sexual things”—in the last 12 months (Kann et al. 2018). Of the nearly 70% of high school students who reported dating in a national survey, 7% were forced to do something sexual in the prior year by someone they dated or went out with—a sexual form of teen dating violence (TDV; Kann et al. 2018). Sexual harassment (SH)—unwelcome conduct of a sexual nature including sexual advances, requests for sexual favors, or other unwanted verbal, nonverbal, or physical conduct of a sexual nature—was also experienced by almost half (48%) of all 7th-12th graders during the prior school year in another national survey, and most of the students affected (87%) reported that the experience had a negative effect on them (Hill and Kearl 2011). Thus, early adolescence—when sexual behavior patterns are developing—provides an opportune time to intervene with youth to reduce their risk of SV perpetration or victimization as they mature and enter intimate relationships.

Only a handful of programs have been shown to be promising or effective for preventing SV among adolescents (Community Preventive Services Task Force 2018; DeGue et al. 2014): Safe Dates (Foshee et al. 2004), Green Dot (Coker et al. 2017), Coaching Boys into Men (Miller et al. 2013), Second Step (Espelage et al. 2013), Expect Respect Support Groups (ERSG; Reidy et al. 2017), and Shifting Boundaries (Taylor et al. 2013). These interventions—most developed to prevent TDV—focus on teaching skills for healthy relationships (including healthy sexuality and sexual consent), empowering positive bystander behavior, promoting social norms that protect against violence, and/or creating protective school environments to reduce rates of SV among middle and high school students. The Centers for Disease Control and Prevention (CDC) has identified each of these approaches as promising for the prevention of SV based on their efficacy in past research (Basile et al. 2016).

Despite these advances in the evidence base, it is increasingly clear that no single program will be sufficient to reduce rates of SV—or other forms of violence—at the population level (DeGue et al. 2016; DeGue et al. 2012). Comprehensive prevention strategies that address risk for SV across the social ecology with multiple, coordinated interventions may have greater potential to change the social and physical contexts that influence risk behavior—in addition to risk and protective characteristics of the individual (DeGue et al. 2016). When designed to address shared risk and protective factors across related health outcomes, comprehensive prevention approaches may also have greater potential to influence multiple forms of violence and health risk behaviors, including SV and TDV, increasing the efficiency of prevention efforts (Wilkins et al. 2014). Yet, to date, no comprehensive prevention strategies for SV or TDV have undergone rigorous evaluation to assess their efficacy for reducing violence risk relative to existing evidence-based, single-program prevention strategies.

To advance this evidence base, CDC developed Dating Matters*®: Strategies to Promote Healthy Teen Relationships* (Teten Tharp 2012; Teten Tharp et al. 2011). In contrast to the existing single-program prevention strategies, Dating Matters is a multicomponent, comprehensive TDV prevention model focused on middle school youth as well as their parents, schools, and neighborhoods. Dating Matters addresses a range of risk and protective factors that impact early adolescents (ages 11–14) across the social ecology, including many factors shared with other forms of violence. Dating Matters youth programs, for example, address several factors associated with both SV and TDV, such as healthy relationship skills, healthy communication skills, consent education, sexual coercion, bullying, SH, dating safety, supporting victims, relationship rights, and getting help. Dating Matters was originally developed to address TDV and prior research suggests it is effective at preventing TDV perpetration and victimization—including a combined measure of physical, sexual, and emotional violence by a current or former dating partner (Niolon et al. 2019). Additional analyses have also found positive effects of Dating Matters on other related outcomes, including physical peer violence, bullying, cyber-bullying, weapon carrying, alcohol and substance abuse, and delinquency (Estefan et al. under review; Vivolo-Kantor et al. 2019). Thus, Dating Matters may have effects on other forms of violence as well, including SV and SH.

Extending past research, the current study examines the effectiveness of the Dating Matters comprehensive TDV prevention model compared to a standard-of-care TDV program on SV and SH victimization and perpetration outcomes among middle school students. While prior research included SV against a dating partner in the assessment of TDV, the current study measures SV and SH exposure inclusive of any victim-perpetrator relationship. Specifically, we hypothesized that students exposed to Dating Matters will report less SV and SH victimization and perpetration over time compared to students in the standard-of-care condition. Although CDC defines SV broadly to encompass a range of nonconsensual sexual acts including rape, sexual coercion, unwanted sexual contact, and SH, we use the term SV in the current study to refer to any physically forced sexual contact and examine non-physical SH separately.

## Method

This study draws from a larger randomized controlled trial (RCT) to evaluate intervention effects on teens’ exposure to SV and SH, as victims or perpetrators, in middle school. All procedures were approved by CDC and local Institutional Review Boards, as well as the Office of Management and Budget (OMB #0920–0941). Additional details on the study methods and sample are available in Niolon et al. (2016); Niolon et al. (2019). A CONSORT diagram is provided in the supplemental materials (Supplemental Fig. 1).

### Design and Sample

Middle schools serving high-risk communities—defined by above average rates of crime and poverty—in four urban areas in the USA were randomly assigned to receive the Dating Matters comprehensive prevention model (DM; *N* = 22) or a standard-of-care intervention (SC; *N* = 24) over four consecutive school years (2012–2016). All assenting students with parental consent (grades 6–8) were surveyed during the school day in fall and spring of each school year. Data were collected from five cohorts of middle school students from 2012 to 2015. Cohorts 1–3 were in 8th, 7th, and 6th grades, respectively, in the 2012–2013 school year. Cohorts 4 and 5 were added as 6th graders in 2013–2014 and 2014–2015, respectively (Niolon et al. 2019). The analytic sample for the current paper includes two full-exposure cohorts (cohorts 3 and 4) who entered the study in 6th grade and completed 8th grade by the end of the study (total sample: 3301; DM: *N* = 1662, SC: *N* = 1639), allowing an opportunity for exposure to all 3 years of youth and parent programs in the DM condition. A total of 6 waves of data are included collected from two cohorts of 6th–8th grade students in the DM and SC schools over four school years (fall 2012 to spring 2016). Each cohort is assessed in the fall and spring of their 6th, 7th, and 8th grade years, for a total of 6 waves for each cohort. Cohorts 3 and 4 were analyzed separately. As cohort 3 started in the first year of implementation and cohort 4 started in the second year of implementation, we anticipated potential cohort effects due to improvements in implementation quality in year 2 and greater potential for school-level effects (e.g., norms change) with school-wide implementation having been in place for 1 year prior. The overall survey participation rate was 79.7%. The sample was majority female (53%) and predominantly black, non-Hispanic (50%) or Hispanic (31%) with a mean age of 11.93 (*SD* = .57). See Supplemental Fig. 1 for the CONSORT diagram, which includes specific information on the sample at each wave.

### Intervention

The Dating Matters comprehensive TDV prevention model (Niolon et al. 2016; Niolon et al. 2019; Teten Tharp 2012; Teten Tharp et al. 2011) includes multiple components operating across the social ecology: (1) classroom-delivered programs for youth in 6th, 7th, and 8th grades; (2) community-based programs for parents of 6th, 7th, and 8th grade youth; (3) TDV prevention training for all school staff; (4) a youth communications program implemented by high school-age brand ambassadors; and (5) community-level activities to build capacity for comprehensive prevention efforts, inform local policy, and use local TDV-related indicator data. The 6th and 7th grade youth programs were developed by CDC for Dating Matters (See https://www.cdc.gov/violenceprevention/intimatepartnerviolence/datingmatters/index.html for more information). In 8th grade, youth receive Safe Dates, an evidence-based TDV prevention program (Foshee et al. 2004). All three youth programs focus on developing healthy relationship skills through developmentally appropriate content that progresses from an emphasis on peer and family relationships in 6th grade to dating partners in 8th grade. The parent programs include an adapted version of the evidence-based Parents Matter! program (6th grade), a CDC-developed program called Dating Matters for Parents (7th grade), and Families for Safe Dates (8th grade) (Forehand et al. 2007; Foshee et al. 2012). The parent programs focus on skills for positive parenting and effective parent-child communication about healthy relationships. Each of the multi-session youth and parent programs provide interactive opportunities for skill building and development of positive norms and behaviors. The multiple programmatic and intervention components of the Dating Matters model were designed to work together to address risk and protective factors for TDV across levels of the social ecology through implementation over all 3 years of middle school (Teten Tharp 2012). All of these programs and components were delivered during each year of implementation. For additional information on the *Dating Matters* model, see www.cdc.gov/violenceprevention/datingmatters.

Schools in the standard-of-care condition received Safe Dates in 8th grade only. Safe Dates is a 10-session, school-based program developed for 8th and 9th graders that has been shown to prevent physical and sexual TDV perpetration and victimization at 4 years follow-up (Foshee et al. 2004). As noted, Safe Dates is also included in the Dating Matters model as the 8th grade youth program; thus, all 8th grade study participants received this program in each year of implementation.

### Measures

#### Sexual Violence

SV victimization and perpetration were assessed with variants of a single item from the AAUW Sexual Harassment Survey (American Association of University Women Educational Foundation 2001) asking whether someone had *in person* “forced them to do something sexual” or whether they had forced someone else to do something sexual in their lifetime (at baseline) or in the last 4 months (at follow-up). Response options were 1 (never), 2 (1–3 times), 3 (4–9 times), and 4 (10 or more times). Raw scores for sexual violence perpetration ranged from 1.03 to 1.15 and victimization ranged from 1.06 to 1.14 across time. These outcomes were modeled as manifest variables, since only one item was available for perpetration and one for victimization. Based on unimputed data, 3% of the students in the analytic sample reported at least one incidence of sexual violence perpetration in middle school and 6% reported at least one instance of sexual violence victimization in middle school.

#### Sexual Harassment

SH was assessed with 6 (victimization) or 7 (perpetration) items asking whether they had engaged in a form SH towards someone else, or vice versa, in their lifetime (baseline) or in the last 4 months (at follow-up). Five items from the AAUW Sexual Harassment Survey (American Association of University Women Educational Foundation 2001) assessed *in person* sexual harassment perpetration and victimization (i.e., unwelcome sexual comments, jokes, or gestures; called them gay or lesbian in a negative way; touched them in an unwelcome sexual way; showed them sexual pictures that they did not want to see; physically intimidated them in a sexual way). Two items from the Growing Up in the Media Survey (Ybarra et al. 2011) assessed whether they had asked someone to do something sexual online when the other person did not want to do it (perpetration) or someone asked them (victimization); or sent a picture text message that was sexual in any way when that person did not want to receive it (perpetration only). Response options were 1 (never), 2 (1–3 times), 3 (4–9 times), and 4 (10 or more times). To focus our statistical tests on structural relationships (group mean differences), we created an item average composite for perpetration and another for victimization. Reliability ranged from .64–.74 for victimization and .73–.83 for perpetration across waves. Raw scores for sexual harassment perpetration ranged from 1.09 to 1.31 and victimization ranged from 1.21 to 1.39 across time. Based on unimputed data, 29% of the students in the analytic sample reported at least one incidence of sexual harassment perpetration in middle school and 47% reported at least one instance of sexual harassment victimization in middle school.

### Statistical Analysis

Prior to analysis, missing data were multiply imputed (100 datasets) using PcAux and programmed in R (Lang et al. 2017). All analysis models used all 100 datasets; parameter estimates are averaged using Rubin’s rules (Rubin 1987; Schafer 1997). Because of the way the data were aligned for analyses and other unplanned reasons (e.g., attrition), there was a high proportion of missing data throughout the survey dataset, typically 65% or more. School-level missing data due to attrition (early exit) and the study’s attrition-responsive recruitment strategy (late entry) and missing data due to student-level processes (nonresponse due to school transfer, absenteeism, refusal, spot missingness within a survey, or unclear/unusable responses) were treated as missing at random (MAR). The MAR assumption is appropriate for a large-scale data collection such as Dating Matters, given the breadth of measures that were included in the overall protocol.

Using the imputed data, we standardized the scale of the outcome variables to a “percent of maximum score” (POMS), ranging from 0 (lowest category endorsed for every item) to 100 (highest category endorsed for every item). Our evaluation approach involved setting equality constraints on the means and testing the effect of these constraints on the overall model. This approach is rendered straightforward when other parameters in the model are minimal. For example, when covariate adjustment is conducted as a pre-analysis step, group means are interpretationally equivalent across constrained solutions; this is not the case when covariate adjustment is conducted within the constrained models (i.e., covariate parameters may change across solutions). To simplify the program evaluation model to feature the covariate-adjusted means, outcomes were first regressed onto a set of covariates (race/ethnicity, age, survey date, guardianship, and witnessing violence). Apart from assignment to treatment condition, the nesting of students within schools was considered a “nuisance feature” of the design. In other words, school identifiers were used in the covariate adjustment stage to correct for non-independence of observations. Residuals taken from this regression and corrected for outliers served as covariate-adjusted outcomes in the analysis model (For details, see Niolon et al. 2019). To preserve the POMS scaling, we selected meaningful zero points for each of the covariates.

We evaluated program effects on SV and SH within biological sex and cohort groups using multiple group structural equation models with a maximum likelihood estimator, using Mplus, version 7.4. For each outcome, we estimated the means at each time point to obtain model fit statistics against which subsequent models were compared. We then placed equality constraints on statistically similar means. This approach balances Type I and Type II errors and is well-suited to simultaneous tests of many hypotheses in complex models (Little and Lopez 1997). Baseline equivalence across all groups constituted the initial constraints, followed by constraints of means within similar “bands” of magnitude. Constrained means were evaluated for statistical separation by means of post hoc Wald tests. Generally, an optimal solution (one in which further constraints resulted in failure to maintain adequate model fit) was found within five attempts. If the chi-square difference tests revealed significant decrement in fit, we inferred a violation of baseline equivalence. When warranted, baseline equivalence was imposed within gender and/or cohort group and the relative model fit was evaluated. Baseline equivalence in the outcome measure was established for all outcome models. See Niolon et al. (2019) for additional details on this approach.

## Results

Analyses across condition and cohort over time are presented below for sexual violence and sexual harassment outcomes.

### Sexual Violence

#### Perpetration

**Fig. 1:**
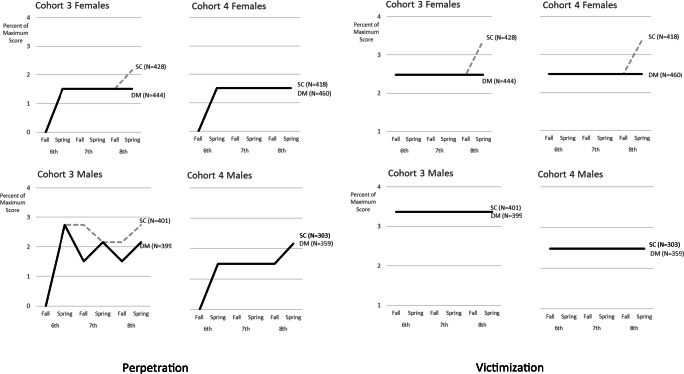
Sexual violence perpetration and victimization across time by sex and cohort. Note. SC = Standard-of-care condition. DM = Dating Matters condition. Percent of Maximum Score (POMS) refers to the maximum possible score given the number of items and response categories in a scale, rather than the maximum observed score. Mean POMS scores have been constrained to appear equal when not significantly different; non-overlapping lines at any time point represent a statistically significant group difference

Significant protective intervention effects were found for Cohort 3 only (see Fig. 1). For Cohort 3 females, effects emerged at the end of 8th grade. For Cohort 3 males, differences were evident at the start of 7th grade and throughout 8th grade. Differences in SV perpetration between DM and SC students across all groups and time points averaged 0.16 POMS (range = 0 to 1.24). The average relative risk reduction across all groups and time points was 6% (range = 0–45%; Fig. 1). By the spring of 8th grade in Cohort 3, the average risk reduction was 13% (range = 0–30%; Suppl. Table 1). Model results and model-estimated means are provided in Table 1.

**Table 1:** Model results and model-estimated means for sexual violence perpetration

Model results: sexual violence perpetration										
Unconstrained				Constrained				Difference		
Chi-square	df	RMSEA	SRMR	Chi-square	df	RMSEA	SRMR	Chi-square	df	*p* value
0.00	0	0.00	0.00	34.30	44	0.00	0.03	34.30	44	0.853
		Rank	Mean			Wald	*p* value			
		1	−0.04		1 v 2	−6.93	0.000			
		2	1.51		2 v 3	−3.03	0.002			
		3	2.75							
Model-estimated means: sexual violence perpetration										
	*N*	Fall 6th	Spring 6th	Fall 7th	Spring 7th	Fall 8th	Spring 8th			
SC Females—Cohort 3	428	−0.04	1.51	1.51	1.51	1.51	2.16			
SC Males—Cohort 3	401	−0.04	2.75	2.75	2.16	2.16	2.75			
DM Females—Cohort 3	444	−0.04	1.51	1.51	1.51	1.51	1.51			
DM Males—Cohort 3	399	−0.04	2.75	1.51	2.16	1.51	2.16			
SC Females—Cohort 4	418	−0.04	1.51	1.51	1.51	1.51	1.51			
SC Males—Cohort 4	392	−0.04	1.51	1.51	1.51	1.51	2.16			
DM Females—Cohort 4	460	−0.04	1.51	1.51	1.51	1.51	1.51			
DM Males—Cohort 4	359	−0.04	1.51	1.51	1.51	1.51	2.16			

Note. *SC* standard-of-care condition, *DM* Dating Matters condition

#### Victimization

Significant protective intervention effects were found for females only, emerging for both cohorts at the end of 8th grade (see Fig. 1). Differences in SV victimization between DM and SC students across all groups and time points averaged 0.09 POMS (range = 0 to .89). The average relative risk reduction across all groups and time points was 3% (range = 0–26%; Fig. 1). By the spring of 8th grade for females, the average risk reduction was 13% (range = 0–26%; Suppl. Table 1). Model results and model-estimated means are provided in Table 2.

**Table 2:** Model results and model-estimated means for sexual violence victimization

Model results: sexual violence victimization										
Unconstrained				Constrained				Difference		
Chi-square	df	RMSEA	SRMR	Chi-square	df	RMSEA	SRMR	Chi-square	df	*p* value
0.00	0	0.00	0.00	44.82	46	0.00	0.03	44.82	46	0.522
		Rank	Mean			Wald	*p* value			
		1	2.48		1 v 2	−3.17	0.002			
		2	3.37							
Model-estimated means: sexual violence victimization										
	*N*	Fall 6th	Spring 6th	Fall 7th	Spring 7th	Fall 8th	Spring 8th			
SC Females—Cohort 3	428	2.48	2.48	2.48	2.48	2.48	3.37			
SC Males—Cohort 3	401	3.37	3.37	3.37	3.37	3.37	3.37			
DM Females—Cohort 3	444	2.48	2.48	2.48	2.48	2.48	2.48			
DM Males—Cohort 3	399	3.37	3.37	3.37	3.37	3.37	3.37			
SC Females—Cohort 4	418	2.48	2.48	2.48	2.48	2.48	3.37			
SC Males—Cohort 4	392	2.48	2.48	2.48	2.48	2.48	2.48			
DM Females—Cohort 4	460	2.48	2.48	2.48	2.48	2.48	2.48			
DM Males—Cohort 4	359	2.48	2.48	2.48	2.48	2.48	2.48			

Note. *SC* standard-of-care condition, *DM* Dating Matters condition

### Sexual Harassment

#### Perpetration

**Fig. 2:**
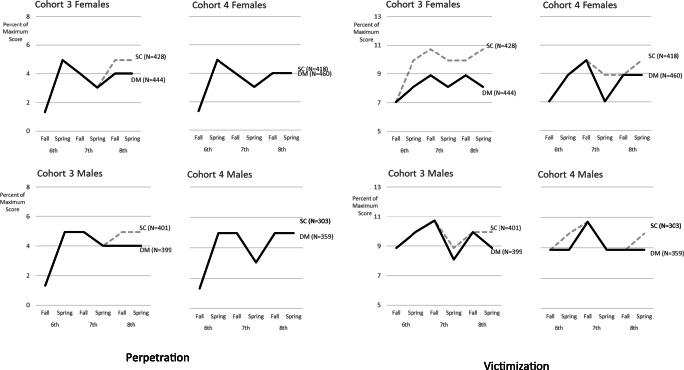
Sexual harassment perpetration and victimization across time by sex and cohort. Note. SC = Standard-of-care condition. DM = Dating Matters condition. Percent of Maximum Score (POMS) refers to the maximum possible score given the number of items and response categories in a scale, rather than the maximum observed score. Mean POMS scores have been constrained to appear equal when not significantly different; non-overlapping lines at any time point represent a statistically significant group difference

**Fig. 3:**
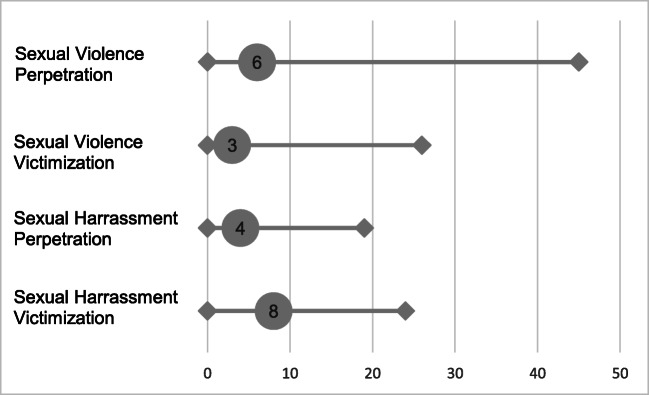
Percent relative risk reduction by outcome across cohorts/time periods (M, range) for Dating Matters vs. standard-of-care. Note*.* Relative risk reduction represents the percent reduction in scores on measures of victimization and perpetration of sexual violence and sexual harassment for the condition relative to the standard-of-care condition. The numbers within the circles represent the average risk reduction for that outcome across the 4 groups (sex by cohort), and the space between the diamonds represent the range of relative risk reduction on that outcome across the four groups

Significant protective intervention effects were found for Cohort 3 only, emerging in the fall of 8th grade and continuing through the spring for both males and females (see Fig. 2). Differences in SH perpetration between DM and SC students across all groups and time points averaged 0.19 POMS (range = 0–.95). The average relative risk reduction across all groups and time points was 4% (range = 0–19%; Fig. 3). By the spring of 8th grade in Cohort 3, the average risk reduction was 10% (range = 0–19%; Suppl. Table 1). Model results and model-estimated means are provided in Table 3.

**Table 3:** Model results and model-estimated means for sexual harassment perpetration

Model results: sexual harassment perpetration										
Unconstrained				Constrained				Difference		
Chi-square	df	RMSEA	SRMR	Chi-square	df	RMSEA	SRMR	Chi-square	df	*p* value
0.00	0	0.00	0.00	55.86	44	0.03	0.04	55.86	44	0.108
		Rank	Mean			Wald	*p* value			
		1	1.34		1 v 2	−6.72	0.000			
		2	3.05		2 v 3	−5.46	0.000			
		3	4.02		3 v 4	−5.16	0.000			
		4	4.96							
Model-estimated means: sexual harassment perpetration										
	*N*	Fall 6th	Spring 6th	Fall 7th	Spring 7th	Fall 8th	Spring 8th			
SC Females—Cohort 3	428	1.34	4.96	4.02	3.05	4.96	4.96			
SC Males—Cohort 3	401	1.34	4.96	4.96	4.02	4.96	4.96			
DM Females—Cohort 3	444	1.34	4.96	4.02	3.05	4.02	4.02			
DM Males—Cohort 3	399	1.34	4.96	4.96	4.02	4.02	4.02			
SC Females—Cohort 4	418	1.34	4.96	4.02	3.05	4.02	4.02			
SC Males—Cohort 4	392	1.34	4.96	4.96	3.05	4.96	4.96			
DM Females—Cohort 4	460	1.34	4.96	4.02	3.05	4.02	4.02			
DM Males—Cohort 4	359	1.34	4.96	4.96	3.05	4.96	4.96			

Note. *SC* standard-of-care condition, *DM* Dating Matters condition

#### Victimization

Significant protective intervention effects were found for all groups (sex and cohort; see Fig. 3). Cohort 3 females saw intervention effects at all time points. Cohort 4 females and Cohort 3 males showed effects in the spring of 7th and 8th grade only. In contrast, Cohort 4 males showed effects in the spring of 6th and 8th grade only. Notably, protective effects were found for all groups in the spring of 8th grade, at the final time point. Differences in SH victimization between DM and SC students across all groups and time points averaged 0.80 POMS (range = 0–2.62). The average relative risk reduction across all groups and time points was 8% (range = 0–24%; Fig. 3; Suppl. Table 1). By the spring of 8th grade, the average risk reduction was 14% (range = 11–24%). Model results and model-estimated means are provided in Table 4.

**Table 4:** Model results and model-estimated means for sexual harassment victimization

Model results: sexual harassment victimization										
Unconstrained				Constrained				Difference		
Chi-square	df	RMSEA	SRMR	Chi-square	df	RMSEA	SRMR	Chi-square	df	*p* value
0.00	0	0.00	0.00	22.65	43	0.00	0.02	22.65	43	0.995
		Rank	Mean			Wald	*p* value			
		1	7.04		1 v 2	−2.70	0.007			
		2	8.10		2 v 3	−2.52	0.012			
		3	8.89		3 v 4	−5.03	0.000			
		4	9.94		4 v 5	−2.66	0.008			
		5	10.73							
Model-estimated means: sexual harassment victimization										
	*N*	Fall 6th	Spring 6th	Fall 7th	Spring 7th	Fall 8th	Spring 8th			
SC Females—Cohort 3	428	7.04	9.94	10.73	9.94	9.94	10.73			
SC Males—Cohort 3	401	8.89	9.94	10.73	8.89	9.94	9.94			
DM Females—Cohort 3	444	7.04	8.10	8.89	8.10	8.89	8.10			
DM Males—Cohort 3	399	8.89	9.94	10.73	8.10	9.94	8.89			
SC Females—Cohort 4	418	7.04	8.89	9.94	8.89	8.89	9.94			
SC Males—Cohort 4	392	8.89	9.94	10.73	8.89	8.89	9.94			
DM Females—Cohort 4	460	7.04	8.89	9.94	7.04	8.89	8.89			
DM Males—Cohort 4	359	8.89	8.89	10.73	8.89	8.89	8.89			

Note. *SC* standard-of-care condition, *DM* Dating Matters condition

## Discussion

*Dating Matters* was associated with significant reductions in SV and SH perpetration and victimization scores in most—but not all—sex/cohort groups by the end of 8th grade relative to a standard-of-care TDV prevention program. However, patterns of findings were inconsistent across cohorts and by sex across outcomes. By the end of middle school, on average, SV perpetration was 13% lower across males and females in Cohort 3 only; SV victimization was 13% lower among females in both cohorts, but not for males, SH perpetration was 10% lower across males and females in Cohort 3 only, and SH victimization was 14% lower across all groups. Despite differences in the patterns of effects over time and across groups, all significant findings were in the hypothesized direction, indicating that Dating Matters had preventive intervention effects on SV and SH over and above the effects of an evidence-based standard-of-care comparison program, Safe Dates.

Notably, no effects were found for Cohort 4 on perpetration of SV or SH. Although the reason is unclear, a similar pattern was seen in prior analyses from this trial examining different outcomes (Estefan et al. 2020) suggesting that Cohort 4 may have experienced or responded to the intervention differently. These findings were inconsistent with our initial expectation that Cohort 4 might demonstrate stronger effects given an additional year of school-level intervention exposure and the potential for improved implementation quality in year 2. Future planned analyses will examine differences in implementation across sites and cohorts to identify possible explanations for these patterns.

Some gender differences were also observed. Fewer effects were found for males overall—in Cohort 3 only for SV and SH perpetration and in both cohorts for SH victimization. However, these effects tended to appear at multiple time points throughout middle school. In contrast, most of the effects for females did not emerge until the end of 8th grade. These patterns could be related to differential developmental onsets of SV and SH behaviors—providing different rates of exposure and opportunity—for males and females over time. The null effects for males on SV victimization were unsurprising given the very low base rates of these experiences among middle school boys.

The most consistent effects were found for SH victimization, with males and females in both cohorts seeing effects at multiple time points during middle school. Thus, the 6th and 7th grade Dating Matters youth programs, and/or other components of the Dating Matters model, may have had stronger effects on SH victimization outcomes than Safe Dates alone. Indeed, the Dating Matters 6th and 7th grade youth programs address SH against peers more directly than Safe Dates, which is focused on skills for healthy dating relationships. It may also be that the effects of Dating Matters were more easily detected on SH victimization outcomes due to higher base rates of these behaviors. Indeed, rates of any perpetration and victimization during middle school in the current study were 3% and 6% for SV compared to 29% and 47% for SH, respectively. Research suggests that some SH behaviors emerge earlier than SV in adolescence and may serve as a developmental precursor (Espelage et al. 2012; Hill and Kearl 2011). Future research could examine that developmental trajectory in this sample as they age into high school.

This study has several notable limitations. First, implementation and data collection in high-risk, urban communities with high mobility and competing school priorities compounded common challenges in conducting school-based prevention research, including school and participant retention, variability in implementation and school characteristics, and completion of consent forms as detailed in Niolon et al. (2019). As such, it is also not known whether these findings are generalizable to rural, suburban, or higher-income, lower-risk communities. Further, the differential effects of intervention exposure or fidelity are, by design, ignored in intent-to-treat analyses and as such may underestimate the effectiveness of the model when delivered as intended. Finally, we relied on self-reported measures of violence perpetration and victimization. Although this is the most common and reliable means of assessing these behaviors, there is inherent potential for misreporting and underreporting.

Despite these limitations, the current study extends prior research examining the effects of Dating Matters to identify promising evidence of effectiveness on SV and SH through middle school. Evidence-based primary prevention strategies for SV and SH remain limited and only a few, including Safe Dates, have evidence of effectiveness for reducing SV and SH in middle school (DeGue et al. 2014). This study provides new evidence for a multi-level comprehensive prevention strategy for SV and SH in middle school, adding substantially to the menu of options available to communities interested in implementing evidence-based sexual violence prevention. In addition, it adds to other evidence showing the effects of Dating Matters on teen dating violence, physical peer violence, bullying, cyber-bullying, weapon carrying, alcohol and substance abuse, and delinquency (Estefan et al. 2020; Niolon et al. 2019; Vivolo-Kantor et al. 2019). These findings suggest that a comprehensive prevention model is more effective than a single-program standard-of-care intervention for preventing SV and SH across all types of relationships, with the potential for cross-cutting effects that address multiple forms of adolescent violence.

Although the absolute difference in violence scores is relatively small, their potential for clinical significance is bolstered by two factors. First, due to the comparative effectiveness design used in this study, the effects demonstrated by Dating Matters are over and above those expected for Safe Dates alone, which has strong evidence of effectiveness for preventing adolescent SV in prior research (Foshee et al. 2004). Second, Dating Matters was designed for implementation during middle school to provide greater opportunity for primary prevention effects, reaching youth as sexual behavior is still developing. As a result, the outcomes in this study are measured through 8th grade, when perpetration and victimization of sexual peer violence and sexual harassment is still at a developmentally lower rate than it will be in later adolescence. As students age and have greater possibility of exposure to SV and SH in high school, any prevention effects that persist should be easier to identify. Given low disclosure rates of SV in middle school when sexual behavior patterns are still developing. As youth mature, exposure to SV and SH, and subsequent disclosure, may increase and these effects may strengthen. Analysis of follow-up data through high school is planned to examine the long-term effects of Dating Matters (For more on plans, see Niolon et al. 2019). In addition to demonstrating the effects of the Dating Matters comprehensive prevention model on these outcomes, this study provides additional evidence of the potential of comprehensive prevention efforts to achieve greater impacts relative to effective single-program models. As communities look to shift towards multicomponent violence prevention approaches, like Dating Matters, evidence that these approaches can achieve improved effectiveness on multiple outcomes is critical to assessing the value of investing in more resource-intensive comprehensive prevention efforts. Further work is needed to understand the implementation costs of these efforts (See Luo et al. under review) and implications for the cost-effectiveness of comprehensive teen dating violence prevention.

## Electronic supplementary material

ESM 1 (PDF 192 kb)

## Footnotes

1 CDC defines sexual violence to include any form of non-consensual sexual act including forced or alcohol/drug facilitated penetration, penetration resulting from pressure or threats (i.e., sexual coercion), intentional sexual touching, non-contact sexual acts (e.g., sexual harassment; Smith et al. 2017). Contact sexual violence includes forced or alcohol/drug facilitated penetration of a victim (i.e., rape), being made to penetrate someone else, sexual coercion, and other unwanted sexual contact.
