# Correction to: Effects of Dating Matters® on Sexual Violence and Sexual Harassment Outcomes among Middle School Youth: a Cluster-Randomized Controlled Trial

**DOI:** 10.1007/s11121-020-01183-7

**Published:** 2020-11-10

**Authors:** Sarah DeGue, Phyllis Holditch Niolon, Lianne Fuino Estefan, Allison J. Tracy, Vi D. Le, Alana M. Vivolo-Kantor, Todd D. Little, Natasha E. Latzman, Andra Tharp, Kyle M. Lang, Bruce Taylor

**Affiliations:** 1 Division of Violence Prevention, National Center for Injury Prevention and Control, Centers for Disease Control and Prevention, 4770 Buford Highway NE, MS-F63, Atlanta, GA 30341 USA; 2 2M Research, LLC, Arlington, TX USA; 3 Institute for Measurement, Methodology, Analysis and Policy, Texas Tech University, Lubbock, TX USA; 4 NORC at the University of Chicago, Chicago, IL USA

**Correction to: Prevention Science**

10.1007/s11121-020-01152-0

The article “Effects of Dating Matters® on Sexual Violence and Sexual Harassment Outcomes among Middle School Youth: a Cluster-Randomized Controlled Trial”, written by Sarah DeGue, Phyllis Holditch Niolon, Lianne Fuino Estefan, Allison J. Tracy, Vi D. Le, Alana M. Vivolo-Kantor, Todd D. Little, Natasha E. Latzman, Andra Tharp, Kyle M. Lang, and Bruce Taylor, was originally published electronically on the publisher’s internet portal on 26 August 2020 without open access. With the author(s)’ decision to opt for Open Choice the copyright of the article changed on 15 October 2020 to © The Author(s) 2020 and the article is forthwith distributed under a Creative Commons Attribution Creative Commons Attribution 4.0 International License, which permits use, sharing, adaptation, distribution and reproduction in any medium or format, as long as you give appropriate credit to the original author(s) and the source, provide a link to the Creative Commons licence, and indicate if changes were made. The images or other third party material in this article are included in the article’s Creative Commons licence, unless indicated otherwise in a credit line to the material. If material is not included in the article’s Creative Commons licence and your intended use is not permitted by statutory regulation or exceeds the permitted use, you will need to obtain permission directly from the copyright holder. To view a copy of this licence, visit http://creativecommons.org/licenses/by/4.0/.

The original article has been corrected.

**Open Access** This article is licensed under a Creative Commons Attribution 4.0 International License, which permits use, sharing, adaptation, distribution and reproduction in any medium or format, as long as you give appropriate credit to the original author(s) and the source, provide a link to the Creative Commons licence, and indicate if changes were made. The images or other third party material in this article are included in the article’s Creative Commons licence, unless indicated otherwise in a credit line to the material. If material is not included in the article’s Creative Commons licence and your intended use is not permitted by statutory regulation or exceeds the permitted use, you will need to obtain permission directly from the copyright holder. To view a copy of this licence, visit http://creativecommons.org/licenses/by/4.0/.

