# Supplementary material for: Effects of Dating Matters® on Sexual Violence and Sexual Harassment Outcomes among Middle School Youth: a Cluster-Randomized Controlled Trial
Source: Prev Sci. 2020 Aug 26;22(2):175–85. doi: 10.1007/s11121-020-01152-0 (PMC7840649; doi:10.1007/s11121-020-01152-0)
Supplement: Supplementary file 1 — (PDF 192 kb) [file 11121_2020_1152_MOESM1_ESM.pdf]

Supplemental Table 1. Estimates of Relative Risk Reduction and Associated Statistical Tests for Sexual Violence and Sexual Harassment Outcomes

			Sexual Harassment								Sexual Violence							
			Perpetration				Victimization				Perpetration				Victimization			
			Cohort 3		Cohort 4		Cohort 3		Cohort 4		Cohort 3		Cohort 4		Cohort 3		Cohort 4	
			F	M	F	M	F	M	F	M	F	M	F	M	F	M	F	M
6th Grade, Spring		Estimate	0.00	0.00	0.00	0.00	18.47	0.00	0.00	10.59	0.00	0.00	0.00	0.00	0.00	0.00	0.00	0.00
		SE					3.38			1.97								
		Wald					5.46			5.39								
		p-value					0.000			0.000								
	95% CI	Lower					11.84			6.74								
		Upper					25.11			14.44								
7th Grade, Fall		Estimate	0.00	0.00	0.00	0.00	17.14	0.00	0.00	0.00	0.00	45.03	0.00	0.00	0.00	0.00	0.00	0.00
		SE					2.44					7.80						
		Wald					7.02					5.77						
		p-value					0.000					0.000						
	95% CI	Lower					12.36					29.74						
		Upper					21.92					60.32						
7th Grade, Spring		Estimate	0.00	0.00	0.00	0.00	18.47	8.82	20.82	0.00	0.00	0.00	0.00	0.00	0.00	0.00	0.00	0.00
		SE					3.38	3.45	3.25									
		Wald					5.46	2.56	6.40									
		p-value					0.000	0.011	0.000									
	95% CI	Lower					11.84	2.05	14.45									
		Upper					25.11	15.59	27.19									
8th Grade, Fall		Estimate	19.05	19.05	0.00	0.00	10.59	0.00	0.00	0.00	0.00	30.32	0.00	0.00	0.00	0.00	0.00	0.00
		SE	3.20	3.20			1.97					7.89						
		Wald	5.95	5.95			5.39					3.84						
		p-value	0.000	0.000			0.000					0.000						
	95% CI	Lower	12.77	12.77			6.74					14.86						
		Upper	25.33	25.33			14.44					45.78						
		Estimate	19.05	19.05	0.00	0.00	24.45	10.59	10.59	10.59	30.32	21.06	0.00	0.00	26.16	0.00	26.16	0.00

		SE	3.20	3.20			3.36	1.97	1.97	1.97	7.89	11.03			6.67		6.67
		Wald	5.95	5.95			7.29	5.39	5.39	5.39	3.84	1.91			3.92		3.92
8th Grade, Spring		*p*-value	0.000	0.000			0.000	0.000	0.000	0.000	0.000	0.056			0.000		0.000
	95% CI	Lower	12.77	12.77			17.87	6.74	6.74	6.74	14.86	-0.56			13.09		13.09
		Upper	25.33	25.33			31.02	14.44	14.44	14.44	45.78	42.68			39.24		39.24

Note: Relative risks of 0.00 reflect the difference between means that were constrained to be equal across DM and SC groups. Because of these constraints, no standard errors or tests of significance are available. F= Female, M = Male.

Supplemental Figure 1. CONSORT diagram for study enrollment, allocation, and data collection and analysis.

Randomized
Middle schools (N=46)

Dating Matters® Comprehensive (DM)

Planning Year & Year 1

23 Schools Randomized

Completed fall data collection: n = 19 schools n = 1326 students
Completed implementation: n = 16 schools*
Completed spring data collection: n = 16 schools* n = 862 students
Dropped before fall data collection:
Not enough consent forms: n = 1 school**
Unspecified reasons: n = 1 school**
School closed: n = 2 schools**
Dropped after fall data collection:
Lacked resources to implement*: n = 1 school (n = 155 students)***

**Two schools lacked resources to implement in Y1 and did not complete spring data collection but stayed in the study and were active Y2 (n= 81 and 44)*

Year 2

18 Schools Retained from Y1, 5 New Schools Randomized

Completed fall data collection: n = 22 schools n = 1672 students
Completed implementation: n = 22 schools
Completed spring data collection: n = 22 schools n = 1321 students
Dropped before fall data collection:
New school, not enough consent forms: n = 1 school **

Year 3

22 Schools Retained from Y2, 2 New Schools Randomized

Completed fall data collection: n = 21 schools* n = 1450 students
Completed implementation: n = 22 schools+
Completed spring data collection: n = 21 schools n = 1097 students
Dropped before fall data collection:
Retained school, lack of freedom to adapt: n = 1 school
Retained school, disliked intervention materials: n = 1 school***
Dropped after fall data collection:
New school, school closed post implementation: n = 1 school (n = 153)***

+One retained school did not participate in fall data collection but implemented and participated in spring.

Year 4

21 Schools Retained from Y3, 0 New Schools Randomized

Completed fall data collection: n = 20 schools n = 611 students
Completed implementation: n = 20 schools
Completed spring data collection: n = 20 schools n = 538 students
Dropped before fall data collection:
Retained school, school closed: n = 1 school

Standard-of-Care (SC)

Planning Year & Year 1

23 Schools randomized

Completed fall data collection: n = 21 schools n = 1568 students
Completed implementation: n = 20 schools*
Completed spring data collection: n = 20 schools* n = 1277 students

Dropped before fall data collection:
Not enough consent forms: n = 1 school**
School closed: n = 1 school**

**One school who lacked resources to implement in Y1 and did not complete spring data collection stayed in the study and was active Y2 (n=95)*

Year 2

21 Schools Retained from Y1, 3 New Schools Randomized

Completed fall data collection: n = 23 schools n = 1706 students
Completed implementation: n = 23 schools
Completed spring data collection: n = 23 schools n = 1343 students
Dropped before fall data collection:
Retained school, unspecified reasons: n = 1 school***

Year 3

23 Schools Retained from Y2, 2 New Schools Randomized

Completed fall data collection: n = 23 schools, n = 1503 students
Completed implementation: n = 23 schools
Completed spring data collection: n = 23 schools, n = 1372 students
Dropped before fall data collection:
Retained school, lacked resources to implement: n = 1 school
Retained school, unspecified reasons: n = 1 school***

Year 4

23 Retained from Y3, 0 New Schools Randomized

Completed fall data collection: n = 23 schools n = 775 students
Completed implementation: n = 22 schools
Completed spring data collection: n = 22 schools n = 595 students
Dropped after fall data collection:
Retained school, failed to implement: n = 1 school (n=56)

Longitudinal Analysis Sample

DM

SCHOOL LEVEL
Omitted
Schools did not contribute data**: n = 5 schools
Schools active less than 2 years***: n = 3 schools n = 459 students
Selected
Schools active a minimum of 2 years: n = 22 schools n = 3495 students
STUDENT LEVEL
Omitted: Cohorts 1, 2, and 5 n = 1833 students
Selected: Cohorts 3 and 4
Full analysis sample: n = 1662 students

SC

SCHOOL LEVEL
Omitted
Schools did not contribute data**: n = 2 schools
Schools active less than 2 years***: n = 2 schools n = 109 students
Selected
Schools active a minimum of 2 years: n = 24 schools n = 3788 students
STUDENT LEVEL
Omitted: Cohorts 1, 2, and 5 n = 2149 students
Selected: Cohorts 3 and 4
Full analysis sample: n = 1639 students

Note: 1. Implementation was whole school, so more students were exposed to the intervention than were included in the trial. Therefore only school numbers are included for completing implementation. 2. When schools dropped out before fall data collection in a given year, student numbers for the lost school could not be estimated because the three grades in school that year had not yet participated in the trial.
